# Face and Body-Based Human Recognition by GAN-Based Blur Restoration

**DOI:** 10.3390/s20185229

**Published:** 2020-09-14

**Authors:** Ja Hyung Koo, Se Woon Cho, Na Rae Baek, Kang Ryoung Park

**Affiliations:** Division of Electronics and Electrical Engineering, Dongguk University, 30 Pildong-ro 1-gil, Jung-gu, Seoul 04620, Korea; koo6190@dongguk.edu (J.H.K.); jsu319@dongguk.edu (S.W.C.); naris27@dongguk.edu (N.R.B.)

**Keywords:** multimodal human recognition, blur image restoration, DeblurGAN, CNN

## Abstract

The long-distance recognition methods in indoor environments are commonly divided into two categories, namely face recognition and face and body recognition. Cameras are typically installed on ceilings for face recognition. Hence, it is difficult to obtain a front image of an individual. Therefore, in many studies, the face and body information of an individual are combined. However, the distance between the camera and an individual is closer in indoor environments than that in outdoor environments. Therefore, face information is distorted due to motion blur. Several studies have examined deblurring of face images. However, there is a paucity of studies on deblurring of body images. To tackle the blur problem, a recognition method is proposed wherein the blur of body and face images is restored using a generative adversarial network (GAN), and the features of face and body obtained using a deep convolutional neural network (CNN) are used to fuse the matching score. The database developed by us, Dongguk face and body dataset version 2 (DFB-DB2) and ChokePoint dataset, which is an open dataset, were used in this study. The equal error rate (EER) of human recognition in DFB-DB2 and ChokePoint dataset was 7.694% and 5.069%, respectively. The proposed method exhibited better results than the state-of-art methods.

## 1. Introduction

Currently, there are several methods of human recognition, including face, iris, fingerprint, finger-vein, and body. However, long-distance face recognition in indoor and outdoor environments is still limited. The human recognition methods can be largely divided into face, body, and iris. However, there are problems with face and iris recognition methods. In these methods, original images can be damaged due to motion blur or optical blur, which is generated when the images of human face or iris are obtained from a long distance. The human recognition performance is significantly degraded due to these types of damages. To solve this problem, the human body is typically used as for long-distance recognition in indoor and outdoor environments.

The data can still contain a blur when human body is used for recognition. However, the human body recognition is less affected than face or iris recognition. There are two methods for human body recognition: gait recognition of an individual and texture and shape-based body recognition, which is based on the still image of a human body. Gait recognition does not exhibit a blur problem. However, the time required for forming the dataset is long because continuous image acquisition is required. Thus, an experiment was conducted indoors for recognition using still images of a human body.

There are disadvantages to human body recognition in an indoor environment. The color of clothes significantly affects the recognition performance. Thus, the human body is divided into two parts to evaluate the recognition performance. In several studies, the body and face have been separated. However, blur restoration of the obtained data has never been performed before.

The method proposed in this study involves restoring the images of human body and face with a blur via a generative adversarial network (GAN). Subsequently, the features of body and face are extracted using a convolutional neural network (CNN) model. The final recognition performance is determined based on the weighted sum and weighted product, which is a score-level fusion approach, using the extracted features.

## 2. Related Work

Previous studies on long-distance human recognition can be divided into human recognition with or without blur restoration, and they can be further divided into single modality-based or multimodal-based methods.

### 2.1. Without Blur Restoration

Single modality-based methods include face recognition, body recognition based on texture, and body recognition based on gait. Several extant studies have been conducted on face recognition. Grgic et al. [1] obtained face data from three designated locations using five cameras. The recognition performance was determined based on principal component analysis (PCA) of the obtained face data. Banerjee et al. [2] used three types of datasets, namely FR_SURV, SCface, and ChokePoint, for the experiment. The recognition was performed through soft-margin learning for multiple feature-kernel combination (SML-MKFC) with domain adaptation (DA). The drawback of face recognition is that facial information is vulnerable to noise, such as blur. There are important features in a face, such as nasal bridge, eyebrow, and skin color, for recognizing an individual. The visibility of facial features is reduced when important features are combined with noise, such as a blur, thereby interfering with face recognition.

Most of the body recognition methods are gait-based, while others are texture and shape-based. For gait-based recognition, Zhou et al. [3] obtained data using two methods of original side-face image (OSFI) and gait energy image (GEI) fusion, as well as enhanced side-face image (ESFI) and GEI fusion. Furthermore, they proceeded with recognition based on PCA and multiple discriminant analysis (MDA). Gait-based recognition is less affected by noise, such a blur, because several images of an individual’s gait are cropped based on the difference image of the background and object. The difference image is compressed into a single image. However, an extensive amount of time and data are required to obtain sufficient gait information. For texture and shape-based body recognition, Varior et al. [4] used the Siamese CNN (S-CNN) architecture. Nguyen et al. [5] obtained image features using AlexNet-CNN and then evaluated the recognition using PCA and support vector machine (SVM). Shi et al. [6] used the S-CNN architecture reported in an extant study [4]. However, they used five convolution blocks. Furthermore, a discriminative deep metric learning (DDML) was used in the study. This method is not significantly affected by a blur because the object’s body information is included. However, the color of clothes worn by the object comprises of a large portion of the body information. Hence, the recognition performance is drastically reduced if the color of the clothes is similar to that of the object, which is being recognized.

Multimodal-based methods are categorized into two types, namely face and gait-based body recognition and face and texture and shape-based body recognition. For face and gait-based body recognition, Liu et al. [7] measured the performance using the dataset obtained by other researchers based on hidden Markov model (HMM) and Gabor features-based elastic bunch graph matching (EBGM). Hofmann et al. [8] used eigenface calculation for face recognition and α-GEI for gait recognition. This method exhibits the same advantages and disadvantages as gait-based body recognition. The common advantage is that it is less affected by a blur because a gait feature is used. The disadvantage is that it requires sufficiently high amount of data with continuous image motion for obtaining the gait image. In a previous study [9], human body and face were separately experimented in indoor environments for face and texture and shape-based body recognition. Visual geometry group (VGG) face net-16 for face and residual network (ResNet)-50 for body were used to obtain the features, and the final recognition performance was evaluated based on a score-level fusion approach using the obtained features. However, the problem with blur still persists when images are obtained in indoor environment. Therefore, in the study [9], only the images without a blur were used by determining the presence of a blur as per the threshold based on the method in the study [10].

### 2.2. With Blur Restoration

A blur is generated due to two main reasons. Motion blur is generated when an object moves, and optical blur is generated when a camera films the object. Thus, researchers improved the images using a deblur method and then proceeded with the evaluation of the recognition performance. Alaoui et al. [11] performed image blurring by applying point spread function (PSF) with the face recognition technology (FERET) database. The images were deblurred with fast total variation (TV)-l1 deconvolution, image features were obtained using PCA, and feature matching was performed with Euclidean distance. Hadid et al. [12] generated a blur using PSF and then proceeded with deblurring based on deblur local phase quantization (DeblurLPQ) and measured the recognition performance. Nishiyama et al. [13] used two types of datasets and generated an arbitrary blur using PSF with the FERET database and face recognition grand challenge (FRGC) 1.0. For blur restoration method, Wien filters or bilateral total variation (BTV) regularization was used. Mokhtari et al. [14] performed face restoration using two methods, namely centralized sparse representation (CSR) and adaptive sparse domain selection with adaptive regularization (ASDS-AR). Face recognition was performed using PCA, linear discriminant analysis (LDA), kernel principal component analysis (KPCA), and kernel Fisher analysis (KFA). Heflin et al. [15] used the FERET database wherein the face area was detected in the blurred image, motion blur and atmospheric blur were measured using a blur point spread function (PSF), and, finally, face deblurring was performed using a deconvolution filter, such as Wiener filter, to evaluate the recognition performance. Yasarla et al. [16] proposed uncertainty guided multi-stream semantic network (UMSN) and performed facial image deblurring. This method involves dividing the facial image region into four semantic networks and deblurring the blurred image and image divided into four regions via a base network (BN). Considering the aforementioned issues of previous researches, we propose a recognition method in which the blur on a body and face is restored using a GAN, and the features of body and face obtained using a deep CNN are used to fuse the matching score.

Although they are not the researches on long-distance human recognition, Peng et al. studied two challenges in clustering analysis, that is, how to cluster multi-view data and how to perform clustering without parameter selection on cluster size. For this purpose, they proposed a novel objective function to project raw data into one space where the projection embraces the cluster assignment consistency (CAC) and the geometric consistency (GC) [17]. In addition, Huang et al. proposed a novel multi-view clustering method called as multi-view spectral clustering network (MvSCN) which could be the first deep version of multi-view spectral clustering [18]. To deeply cluster multi-view data, MvSCN incorporates the local invariance within every single view and the consistency across different views into a novel objective function. They also enforced and reformulated an orthogonal constraint as a novel layer stacked on an embedding network.

Table 1 shows the summary of this study and previous studies on person recognition using surveillance camera environment.

## 3. Contribution of Our Research

Our research is novel in the following four ways in comparison to previous works:

- This is the first approach for multimodal human recognition by blur restoring the face and body images using GAN.
- Different from previous work [9], the presence of a blur was determined based on a focus score method in which blur restoration was applied via GAN for image in case that input image was determined as blur existence. The error was reduced, when compared to that without proposed focus score method and GAN.
- The structural complexity was reduced by separating the network for blur restoration and the CNN for human recognition. In addition, the processing speed is usually faster when one image of face and body is restored at simultaneously via GAN. However, our blur restoration proceeded separately through GAN because face images exhibit detailed information, and the generation of a blur exhibits different tendencies in face and body images.
- We make Dongguk face and body database version 2 (DFB-DB2), trained VGG face net-16 and ResNet-50, and GAN model for deblurring available by other researchers through [19] for fair comparisons.

## 4. Proposed Method

### 4.1. System Overview

**Figure 1:**
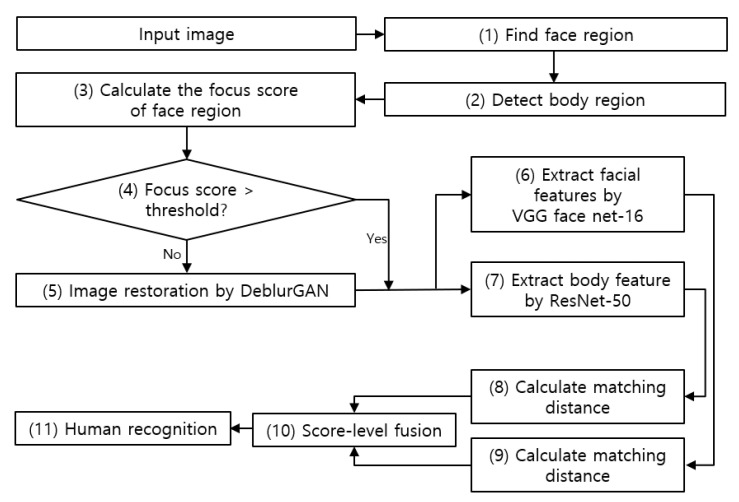
Overall procedure of proposed method.

Figure 1 shows the overall configuration of the system proposed in this study. A face image is obtained from the original image acquired in an indoor environment (step (1) in Figure 1). A body image is obtained from the original image excluding the face image (step (2) in Figure 1). The focus score of the face image is calculated (step (3) in Figure 1). An image exhibiting a focus score value of less than the threshold (step (4) in Figure 1) undergoes restoration using DeblurGAN (step (5) in Figure 1) and is combined with images exhibiting a focus score value that is greater than or equal to the threshold. The restoration of body image via DeblurGAN is conducted in the same manner. Image features of face and body are extracted by applying a CNN model to the image combined from the restored face and body images and the image with a focus score greater than or equal to the threshold (step (6) and (7) in Figure 1). The authentic/imposter matching distance is calculated using the feature vectors obtained above (step (8) and (9) in Figure 1). The score-level fusion is conducted using the matching distance (step (10) in Figure 1). The weighted sum and weighted product methods were for the score-level fusion in this study. The final recognition rate was measured using score-level fusion (step (11) in Figure 1).

### 4.2. Structure of GAN

A general description of a GAN is provided in this section. GAN consists of two networks, namely generator and discriminator. Generator aims to generate a fake image similar to a real image by considering Gaussian random noise as an input, whereas discriminator aims to find the fake image by discriminating the real image from the fake image generated by the generator. Therefore, a discriminator is trained to easily discriminate real and fake images, while a generator is trained to ensure that a fake image is close to the real image to the maximum possible extent. However, it is difficult to control the desired output for vanilla GAN because the input corresponds to Gaussian random noise.

First, cycle-consistent adversarial networks (CycleGAN) [20] were used. Unlike the existing GAN models, a CycleGAN does not distinguish between an input image and a target image. It uses a reference image as an input that is expected to be the result of input image and output image. There are two types of generators in CycleGAN, namely U-Net [21] architecture and residual blocks. The generator used in this study exhibits a residual block architecture [20]. One of the characteristics of a CycleGAN is the cycle-consistency loss. For example, if an input image X has generated an output Y through a generator, the output Y goes through the generator again to generate X’. The cycle-consistency loss refers to calculating the difference between X and X’.

Second, Pix2pix [22] was used. Pix2pix is a GAN applied with the concept of a conditional GAN (CGAN) mode. The generator of Pix2pix is similar to that of U-Net [21]. Unlike U-Net, skip-connection is applied between the encoder and decoder because a blur problem occurs due to the loss of image details when the size of the image is enlarged and then reduced. Furthermore, DeblurGAN [23] uses the input image and target image of a CGAN as an input. However, it exhibits a very different architecture. The architecture of the generator in DeblurGAN consists of two convolutional blocks, 9 residual blocks, and two transposed convolution blocks. Each convolution block contains instance normalization layer [24] and rectified linear units (ReLU) layer, as shown in Table 2. Instance normalization [24] is also referred as contrast normalization. ReLU layer serves as an activation function in residual blocks. The loss function of DeblurGAN uses adversarial loss and content loss. The total loss of the two loss functions can be calculated using Equation (1) as follows:

(1) $$L_{total}\;=L_{Adv}+\lambda L_{Cont}.$$

First, adversarial loss ($L_{Adv}$) can be explained as follows. The adversarial loss discerns the blurred image restored via a generator by using a discriminator. In this case, the loss is considered as optimal when the difference between the loss discerned by the discriminator and the threshold value 1 is close to 0. Thus, $L_{Adv}$ used in DeblurGAN is represented in Equation (2) as follows:

(2) $$L_{Adv}=\;\sum_{k=1}^{N}-D_{\theta }\left(G_{\theta }\left(I_{B}\right)\right).$$

In Equation (2), N denotes the number of images, $D_{\theta }$ denotes the discriminator network, $G_{\theta }$ denotes the generator network, and $I_{B}$ denotes a blurred image. As specified in DeblurGAN [23], Wasserstein GAN-gradient penalty (WGAN-GP) [25] was used for the adversarial loss. Next, $L_{Cont}$ is explained in Equation (3).

(3) $$L_{Cont}=\;\frac{1}{X_{n,m}Y_{n,m}}\sum_{k=1}^{X_{n,m}}\sum_{j=1}^{Y_{n,m}}{\left(\emptyset_{n,m}{\left(I_{S}\right)}_{n,m}-\emptyset_{n,m}{\left(G_{\theta }\left(I_{B}\right)\right)}_{n,m}\right)}^{2}.$$

**Figure 2:**
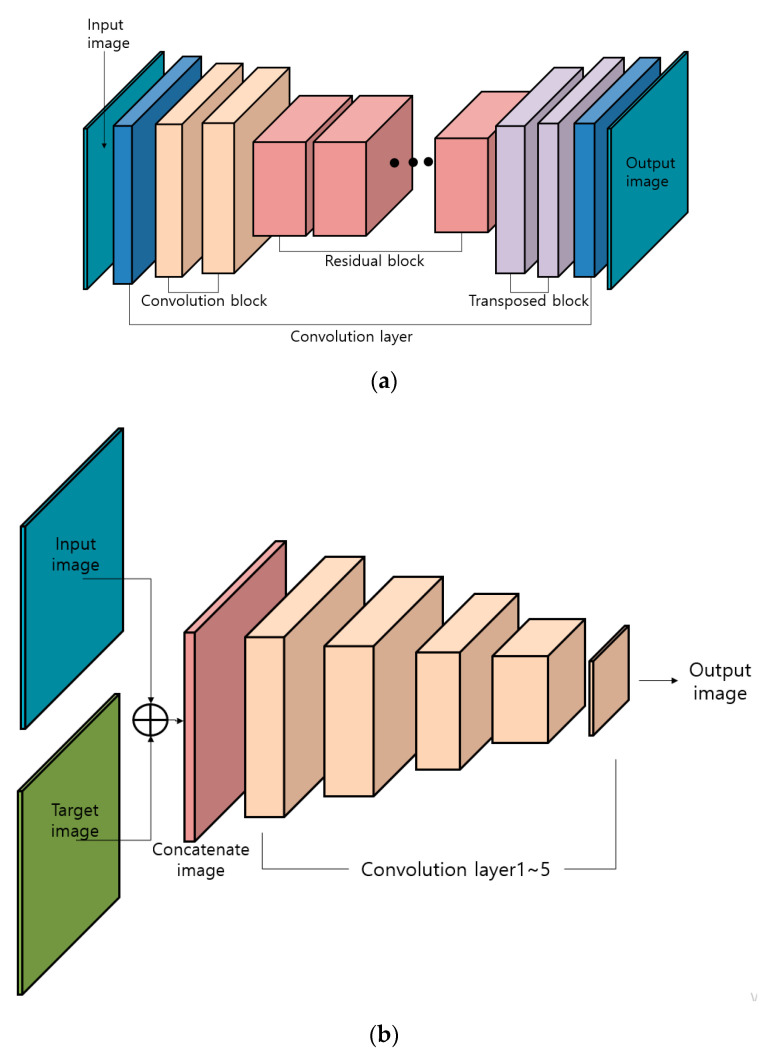
Architecture of DeblurGAN (**a**) Generator in DeblurGAN and (**b**) Discriminator in DeblurGAN.

With respect to content loss, either L1 or mean absolute error (MAE) loss or L2 or mean squared error (MSE) loss can be selected. However, perceptual loss was selected for the content loss of DeblurGAN. The perceptual loss of DeblurGAN can be distinguished by the difference between the restored image and target image obtained through conv3.3 features maps of VGG-19 pretrained with ImageNet. In Equation (3), $X_{n,m}\;and\;Y_{n,m}$ are the size of a feature map, and $\emptyset_{n,m}$ is the feature map obtained from the *m*th convolutional layer. Furthermore, $I_{S}$ is the target image for restoring the blurred image [23]. Table 2 and Table 3 summarize the architecture of the generator and discriminator in DeblurGAN. Figure 2a,b denote the architecture of a generator and discriminator in DeblurGAN, respectively.

### 4.3. Structure of Deep Learning (VGG Face Net-16 and ResNet-50)

The face and body images restored with DeblurGAN used VGG face net-16 and ResNet-50. In our previous research [9], we compared the recognition accuracies by VGG face net-16 and ResNet-50 with those by other CNN architectures on the custom-made Dongguk face and body database (DFB-DB1) whose acquisition environments including scenario and cameras were same to those of DFB-DB2 used in our research. According to the experimental results, VGG face net-16 and ResNet-50 outperform other CNN architectures, and we adopt these CNN models in our research. A pretrained model was used for two types of CNN models, which were fine-tuned based on the characteristics of the dataset used in this study.

The VGG face net-16, which was used for face images, consists of convolution filters and neural network. Specifically, it consists of 13 convolutional layers, five pooling layers, and three fully connected layers. The CNN pretrained model used in this study was trained with Labeled faces in the wild [26] and YouTube faces [27]. The size of the image restored with GAN corresponded to 256 × 256, and it was resized to 224 × 224 for using VGG face net-16 for fine-tuning. The resized image undergoes convolution calculation through the convolutional layer. The calculation is as follows: output = (W − K + 2P)/S + 1. Here, W denotes the width and height of an input, K denotes the size of a convolutional layer filter, P denotes padding, and S denotes stride. For example, if a 224 × 224 image has convolution filter with K = 3, P = 0, and S = 1, then the output is (224 − 3 + 0)/1 + 1, i.e., 222.

There are many types of ResNet based on the number of convolutional layers. As the number of layers increase, the feature map of body images becomes smaller, and thereby causing a vanishing or exploding gradient problem. Thus, a shortcut is used for the ResNet architecture to avoid such a problem. In the shortcut, the input X goes through three convolutional layers and performs convolution calculation three times. If input X that has completed the convolution calculation is termed as F(x), then the shortcut is the sum of the features, or F(x) + X, which is then used as an input for the next convolutional layer. To reduce the convolution calculation time, 1 × 1, 3 × 3, and 1 × 1 convolutional layers were used as opposed to two 3 × 3 convolutional layers. This is termed as the bottleneck architecture wherein 1 × 1 in the front reduces the dimension of the input image, while the 1 × 1 in the back enlarges the dimensions.

## 5. Experimental Results and Analysis

### 5.1. Experiments for Database and Environment

**Figure 3:**
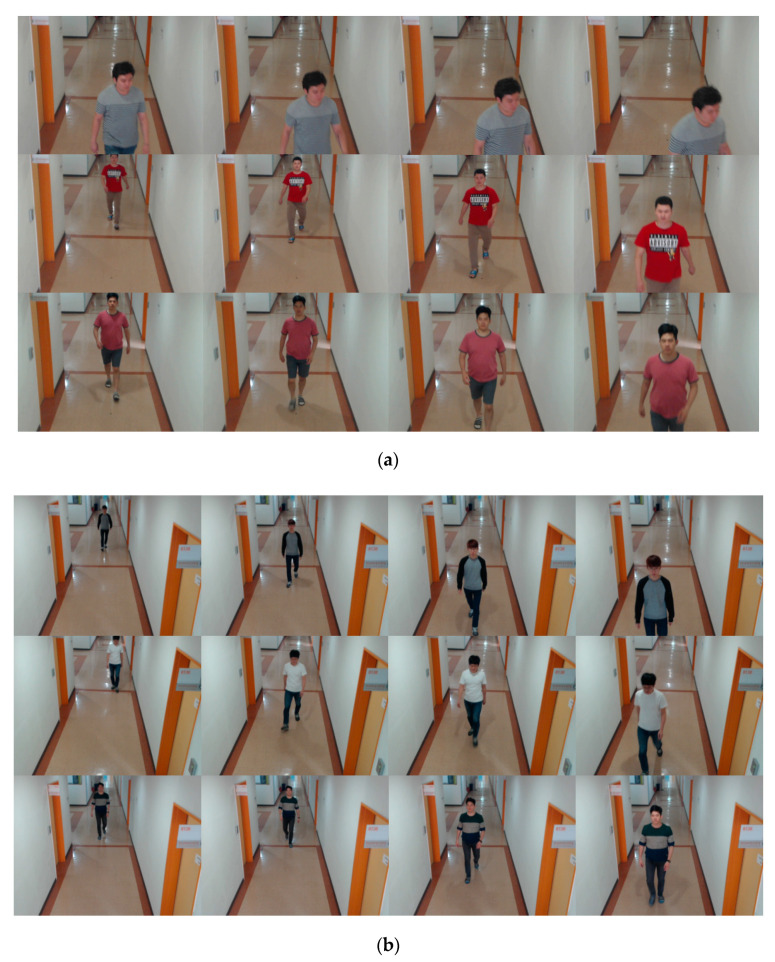
Representative Dongguk face and body dataset version 2 (DFB-DB2) images captured by (**a**) Logitech BCC950 camera and (**b**) Logitech C920 camera.

Two types of cameras were used in this study to acquire the DFB-DB2. The cameras were Logitech BCC950 [28] and Logitech C920 [29]. The cameras were also used for Dongguk face and body dataset version 1 (DFB-DB1). There was no difference in the scenario used for DFB-DB2 and DFB-DB1 in the study [9]. Furthermore, the DFB-DB1 only consists of images above the threshold based on the method of an extant study [10]. However, the DFB-DB2 used in this study included images below the threshold that were restored with DeblurGAN. Figure 3 shows the scenario of the images with respect to DFB-DB2. In the figure, (a) shows the images acquired via the Logitech BCC950 camera, whereas (b) shows those acquired via the Logitech C920 camera.

Table 4 summarizes the details of face and body images of two databases, namely DFB-DB2 and ChokePoint dataset [30], used in this study. Two-fold cross validation was applied to both databases and each dataset was divided into sub-dataset 1 and 2. For example, if sub-dataset 1 is used for training, then sub-dataset 2 is used for testing. Furthermore, if sub-dataset 2 is used for training, then sub-dataset 1 is used for testing to evaluate the performance.

**Figure 4:**
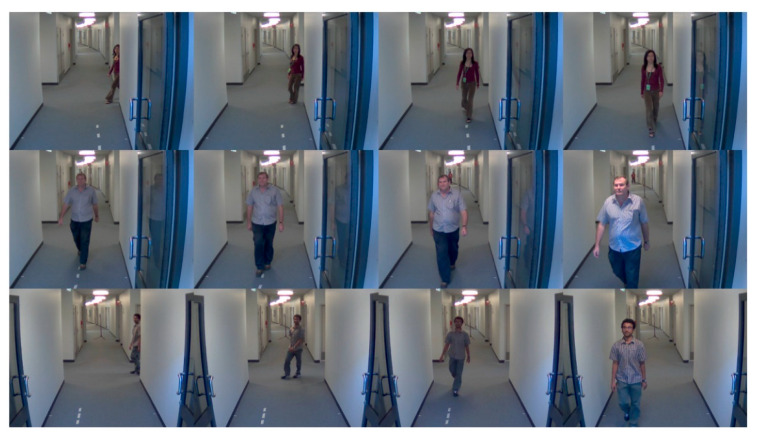
Example images for ChokePoint dataset.

The ChokePoint dataset is provided at no cost by National ICT Australia Ltd. (NICTA) and consists of Portal 1 and 2. Portal 1 contains 25 individuals (19 males and 6 females), and Portal 2 contains 29 individuals (23 males and 6 females). A total of three cameras were used from six locations to constitute the dataset. The dataset of the study [9] was maintained. Furthermore, the images considered exhibit a blur, based on the threshold value in an extant study [10], were restored with DeblurGAN and included for evaluating the recognition performance. Figure 4 shows the examples of the ChokePoint dataset.

### 5.2. Training DeblurGAN and CNN Models

#### 5.2.1. DeblurGAN Model Training Process and Results

Blur image and clear image were distinguished for training DeblurGAN based on the focus score threshold value [9]. The values below the threshold were set as test images for DeblurGAN; the focused image exhibiting a value greater than or equal to the threshold was used as a reference image. Pytorch version of DeblurGAN [31] was used for the program. All the images for training and testing DeblurGAN were resized to 256 × 256. The learning rate was 0.0001, and the batch size was 1 for training DeblurGAN.

#### 5.2.2. CNN Model Training Process and Results

After performing image deblurring with DeblurGAN, face images were trained with VGG face net-16 [32] and body images were trained with ResNet-50 [33]. The number of data points for training each deep CNN model was insufficient, thus the number of data points was increased via data augmentation for training.

**Figure 5:**
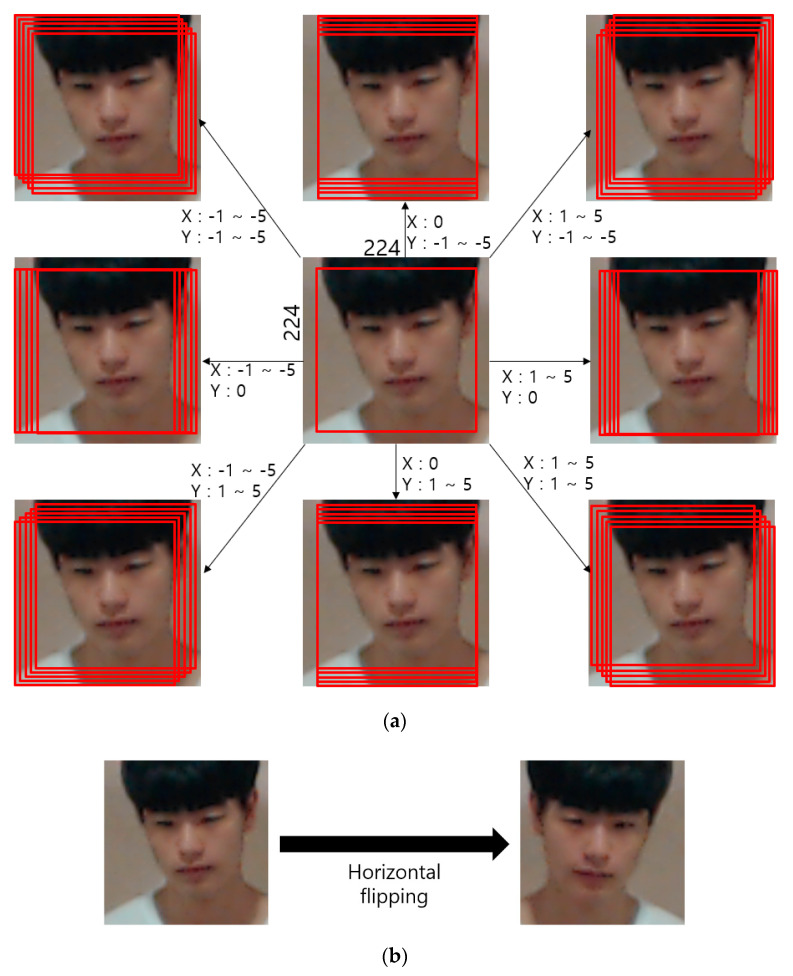
Data augmentation method involving (**a**) image translation and cropping and (**b**) horizontal flipping.

As shown in Table 4, data augmentation was performed only in the training data, whereas the original non-augmented data were used as test data. The number of test data points for the DFB-DB2 is less than that of the ChokePoint dataset, which is an open dataset, and therefore center image crop was performed during augmentation. The cropped image was applied with image translation and cropping for five pixels in top, bottom, left, and right directions. Furthermore, the image was horizontally flipped (mirroring). The training data that was processed accordingly included 440,000 augmented images from sub-datasets 1 and 2. For the ChokePoint dataset, after performing center image crop, image translation and cropping were applied for two pixels in top, bottom, left, and right directions. Furthermore, horizontal flipping was applied to obtain images that were magnified by 50 times. The sub-datasets 1 and 2 in Table 4 include a total of 1.03 million augmented images. Figure 5 shows the data augmentation method used in this study.

Given that VGG face net-16 is pretrained with Oxford face database, it was appropriately fine-tuned for the characteristics of the images in DFB-DB2. Furthermore, ResNet-50 also uses the pretrained model, and thus was appropriately fine-tuned for the characteristics of the image database used in this study. The learning rate was 0.0001, and the batch size was 20 for the training of VGG face net-16 and 15 for the training of ResNet-50.

**Figure 6:**
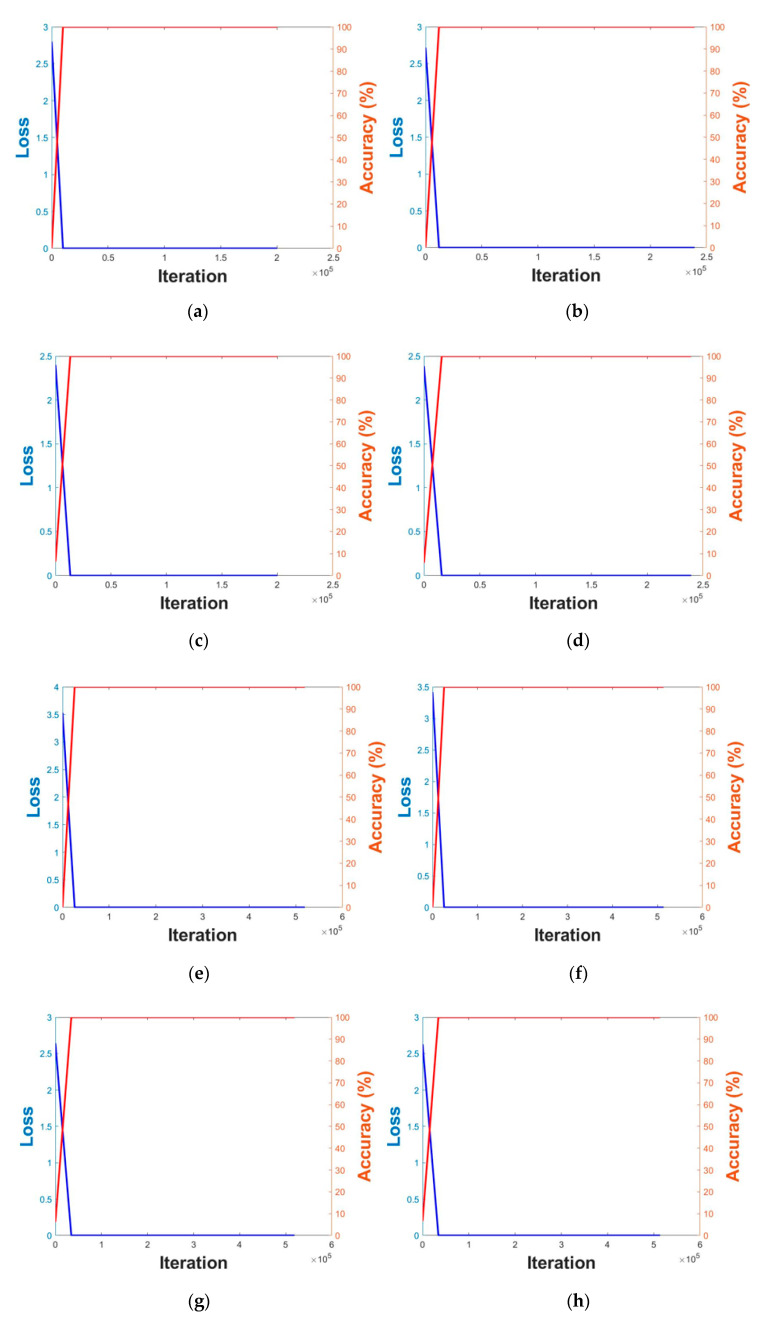
Plots depicting training loss and accuracy of DFB-DB2 ((**a**)–(**d**)) and ChokePoint dataset ((**e**)–(**h**)). Visual geometry group (VGG) face net-16 was used in the case of (**a**,**e**), the 1st fold was used for (**b**,**f**), the 2nd fold ResNet-50 was used in the case of (**c**), 1st fold in the case of (**g**), and the 2nd fold in the case of (**d**,**h**).

Figure 6 illustrates the plots of the loss-accuracy of the training CNN model for trained face and body images. The specifications of the computer used for the experiment are as follows: CPU Intel(R) Core(TM) i7-6700 CPU @ 3.40 GHz, 16 GB RAM, NVIDIA GeForce GTX 1070 graphic card, and CUDA version 8.0.

### 5.3. Testing Results from DeblurGAN and CNN Model

For comparing the original image and deblurred image during the deblurring process, signal-to-noise ratio (SNR) [34], peak signal-to-noise ratio (PSNR) [35], and structural similarity (SSIM) [36] can be used. However, the aforementioned methods, such as SNR, PSNR, and SSIM, cannot be compared with the proposed method because the blur or noise in the blurring images used in this study was naturally generated during the acquisition of the data as opposed to artificial generation of blur or noise in the original image.

#### 5.3.1. Testing with CNN Model for DFB-DB2

Two-fold cross validation was performed to test the training CNN model. For a face image, 4096 features were obtained from the 7th fully connected layer of VGG face net-16. For a body image, 2048 features were obtained from the average pooling layer of ResNet-50. Given the features obtained from the CNN model, the image feature geometric center was calculated by using the Euclidean distance to determine the gallery image. The authentic and imposter distance was calculated by finding the normalized Euclidean distance between the gallery image and other probe images. The distance was used to calculate the equal error rate (EER).

##### Ablation Study

The performance of DFB-DB2 was compared with or without DeblurGAN. Here, “without DeblurGAN” means that both the procedures of focus score checking and DeblurGAN were not operated, whereas “with DeblurGAN” represents that both the procedures of focus score checking and DeblurGAN were adopted. The same DFB-DB2 and ChokePoint dataset were used for the experiment, while VGG face net-16 and ResNet-50 were used for the CNN model. The values in Table 5 and Table 6 show that the recognition performance was improved after using DeblurGAN because there was a reduction in the number of changes in pixels between the original image and image generated after using DeblurGAN.

**Figure 7:**
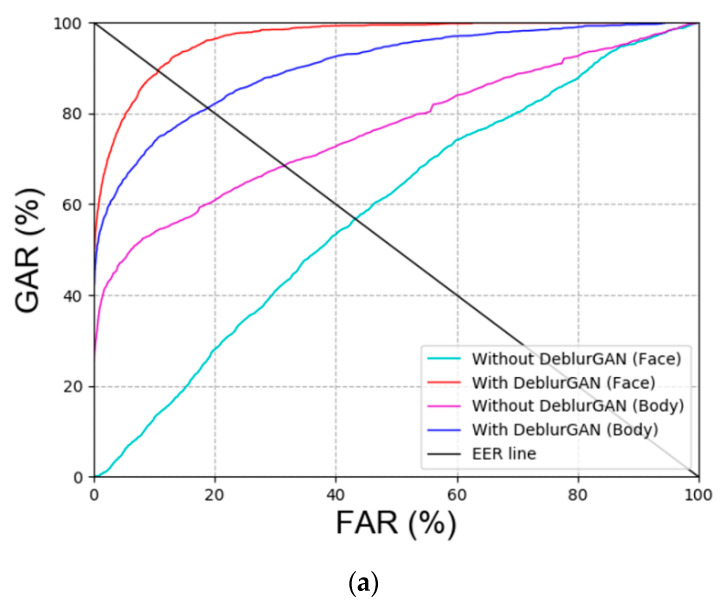

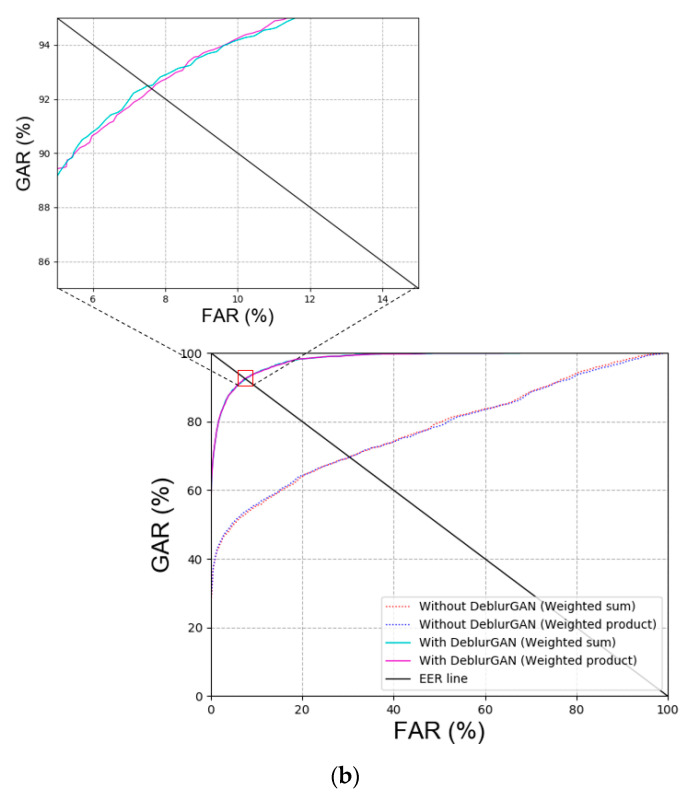
Receiver operating characteristic (ROC) curves with or without DeblurGAN performance of DFB-DB2 (**a**) face and body recognition result and (**b**) score-level fusion result.

As shown in Figure 7, the performance of ‘with DeblurGAN (Face)’ and ‘with DeblurGAN (Body)’ was improved. Face and body refer to face images and body images, respectively. Based on the score-level fusion approach, the weighted sum method exhibited a better performance than the weighted product method.

##### Comparison between Previous Method and Proposed Methods

**Figure 8:**
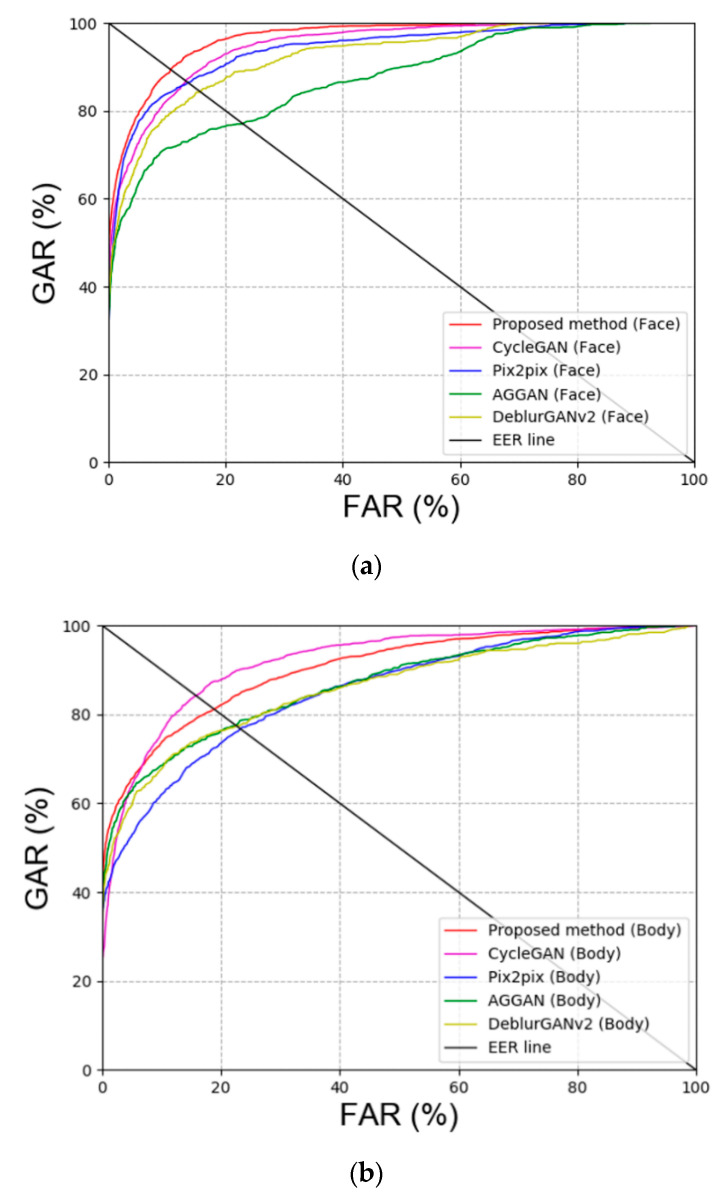

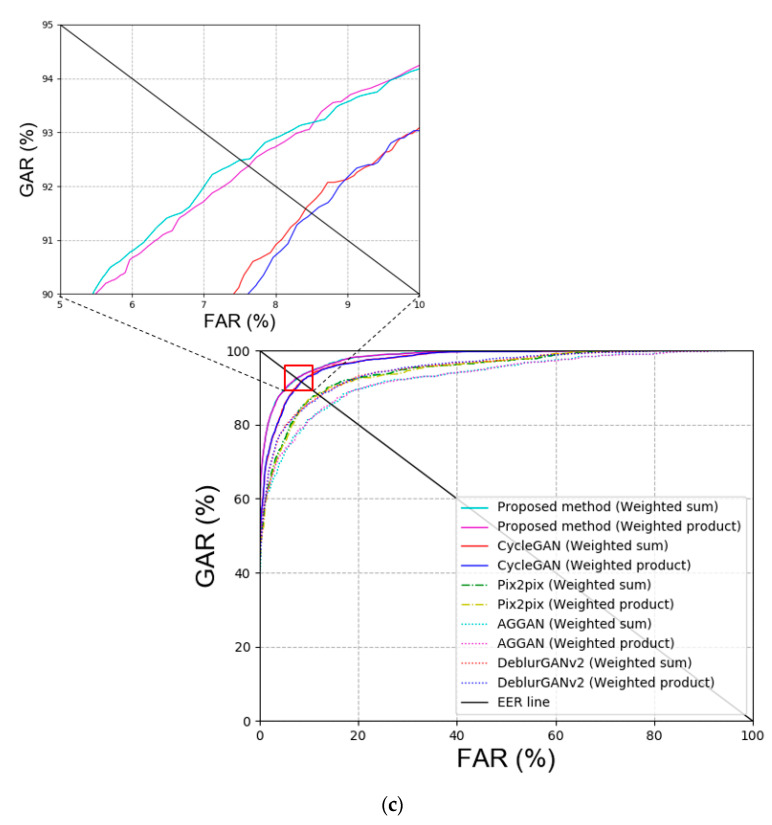
ROC curves via proposed and other GAN methods in DFB-DB2 (**a**,**b**) face and body image recognition results and (**c**) score-level fusion result.

First, blur restoration is performed using other GAN methods besides DeblurGAN, which was proposed in this study for comparison. Specifically, CycleGAN [20], Pix2pix [22], attention-guided GAN (AGGAN) [37,38], and DeblurGAN version 2 (DeblurGANv2) [39] were used for GAN models. Table 7 and Figure 8 show the comparison results of GAN for DFB-DB2, and our method outperforms the state-of-the-art methods. As shown in Table 7, the recognition performance of CycleGAN, which restored the body image in DFB-DB2, was outstanding because DeblurGAN is a CGAN type method wherein the input image and target image are paired. However, when the target image is composed in this study, only the image that is similar to the input image is used for restoration. Therefore, the background, texture of clothes, and the individual’s gait can be different, and this makes the restoration more difficult.

**Figure 9:**
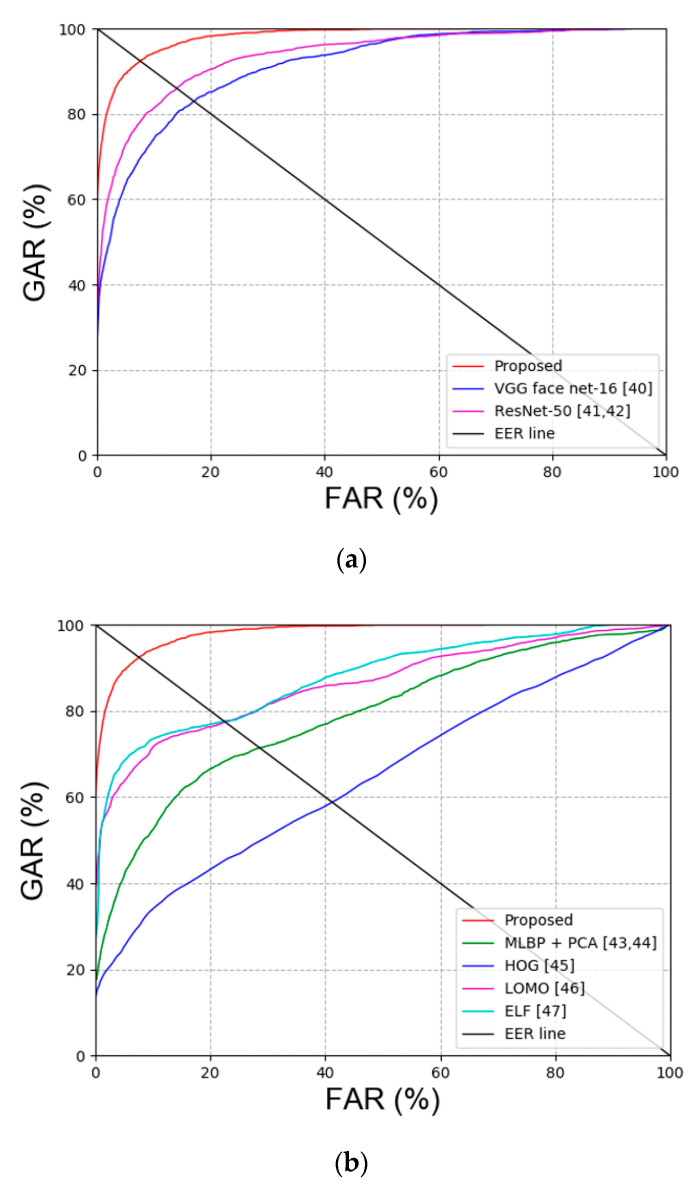
ROC curves obtained via comparing proposed and the state-of-art-methods. (**a**) Face image recognition results and (**b**) face and body image recognition results.

Second, the experiment was conducted to compare face and face and body recognition. The experiment to compare face recognition was conducted with VGG face net-16 [40] and ResNet-50 [41,42]. Multi-level local binary pattern (MLBP) + PCA [43,44], histogram of gradient (HOG) [45], local maximal occurrence (LOMO) [46] and ensemble of localized features (ELF) [47] were used for the experiment to compare face and face and body recognition. Table 8 summarizes the comparison results of face recognition, and Table 9 summarizes the comparison results of face and face and body recognition. Figure 9 shows the receiver operating characteristic (ROC) curve of the results in Table 8 and Table 9.

**Figure 10:**
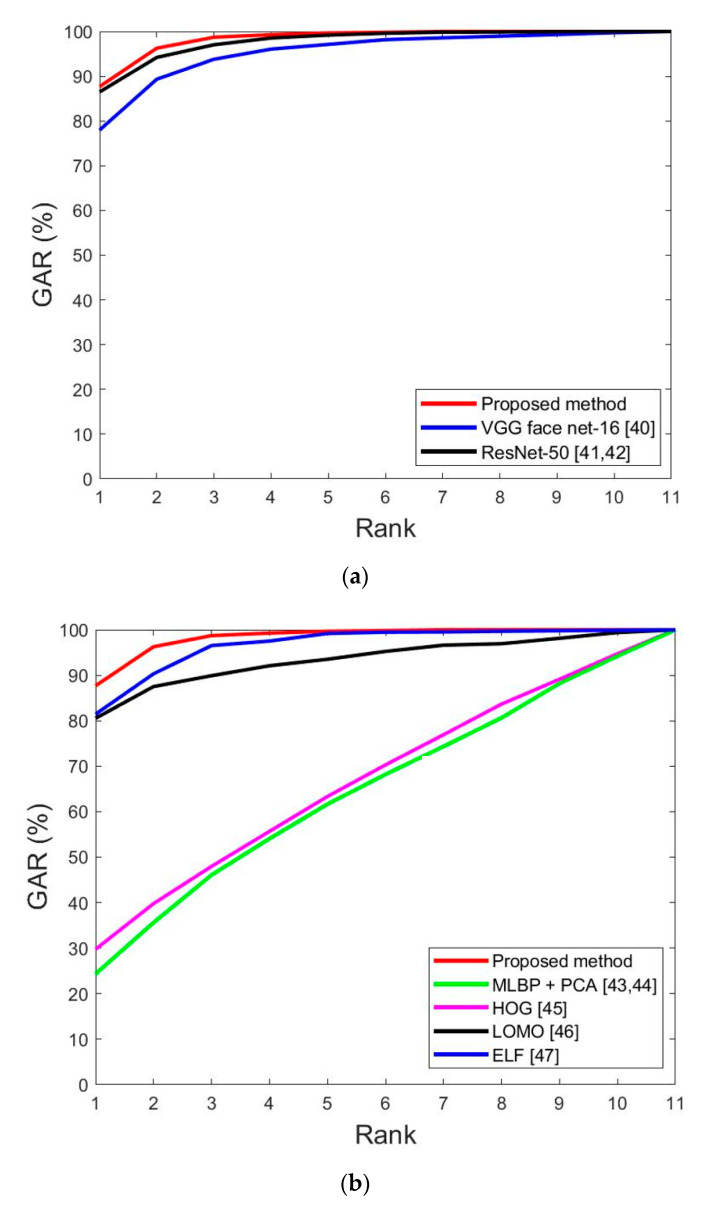
Cumulative match characteristic (CMC) curves of the proposed and previous methods on DFB-DB2. (**a**) Face image recognition results by the proposed and previous methods and (**b**) face and body image recognition results via the proposed and previous methods.

Third, the accuracy of recognition was evaluated via the cumulative match characteristic (CMC) curve. Figure 10 shows the comparison results of the proposed method and methods in Table 8 and Table 9. The horizontal axis corresponds to the rank, and the vertical axis corresponds to the genetic acceptance rate (GAR) accuracy for each rank. Table 4 shows that the DFB-DB2 consists of 11 individuals, as shown in Figure 10.

**Figure 11:**
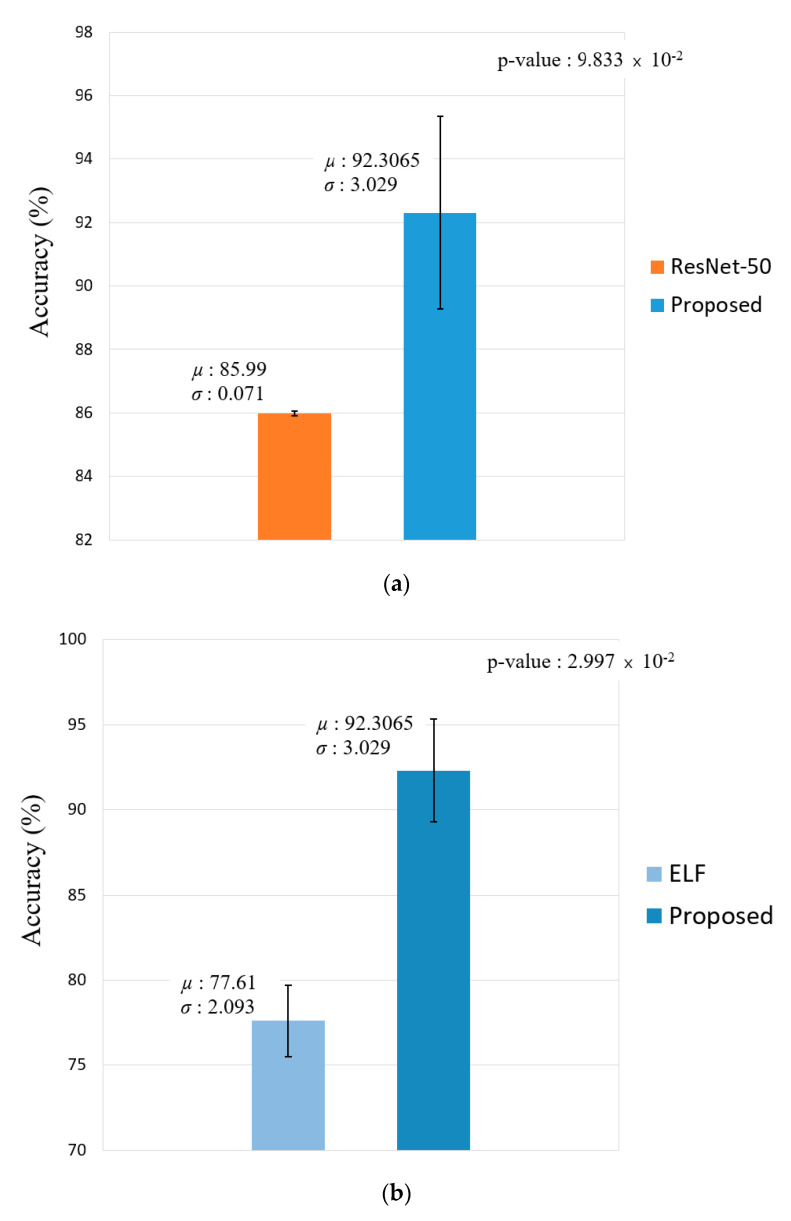
T-test performance of our proposed method and the second-best model in terms of average accuracy. (**a**) Comparison of the proposed method and ResNet-50 and (**b**) comparison of the proposed method and ensemble of localized features (ELF).

Figure 11 shows the difference in the performance by measuring the Cohen’s d-value and t-test results of face recognition and face and body recognition and comparisons with the proposed method. With respect to face recognition, the difference in the Cohen’s d-value between the proposed method and ResNet-50 [41,42] was 2.95. This significantly exceeds the effect size of 0.8 and is thus high. The p-value of the t-test is approximately 0.098, which differs from the proposed method by 99.902%. With respect to face and body recognition, the Cohen’s d-value and t-test results were measured for the ELF [47] that exhibited the second-best performance when compared to that of the proposed method with a Cohen’s d-value of 5.65. This exhibited a large effect size, and the t-test exhibited a difference of 99.97%.

**Figure 12:**
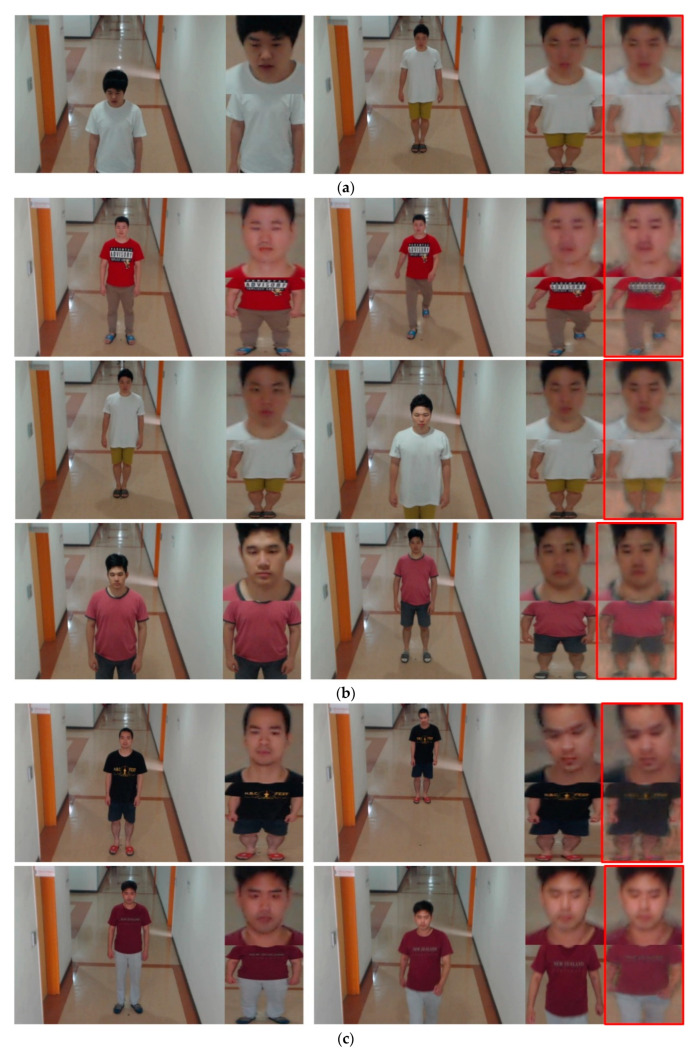
Cases of false acceptance (FA), false rejection (FR), and correction recognition (**a**)–(**c**) in DFB-DB2. (**a**) FA cases, (**b**) FR cases, and (**c**) cases of correct recognition.

The false acceptance ratio (FAR), false rejection ratio (FRR), and correct case of the previous experimental results are analyzed in the plots. Figure 12 illustrates different cases, in which the image on the left corresponds to the enrolled image, and the image on the right corresponds to the probe image. The portion in the red box of the image on the right is restored via DeblurGAN.

#### 5.3.2. Class Activation Map

**Figure 13:**
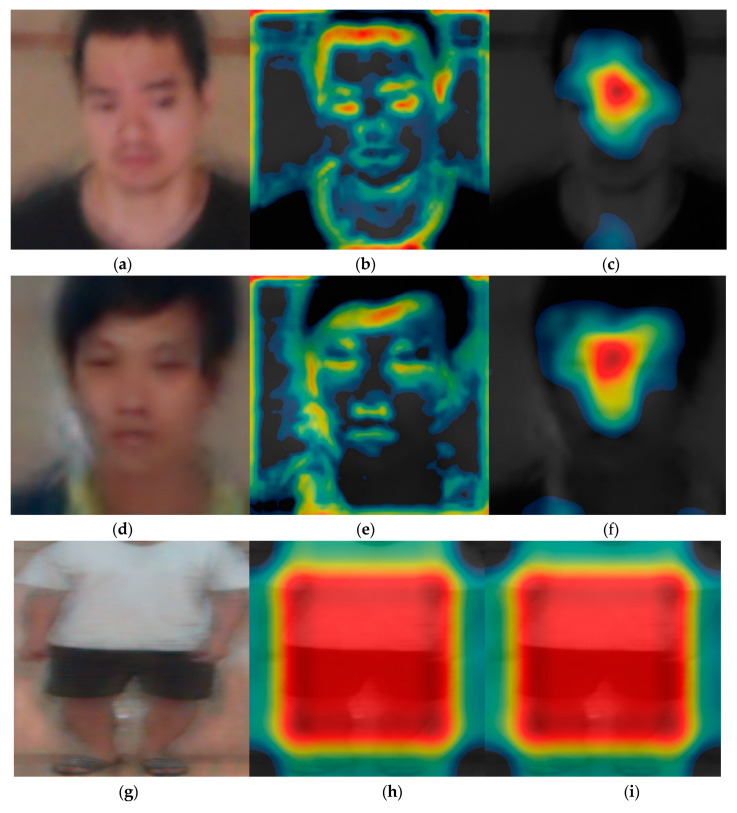

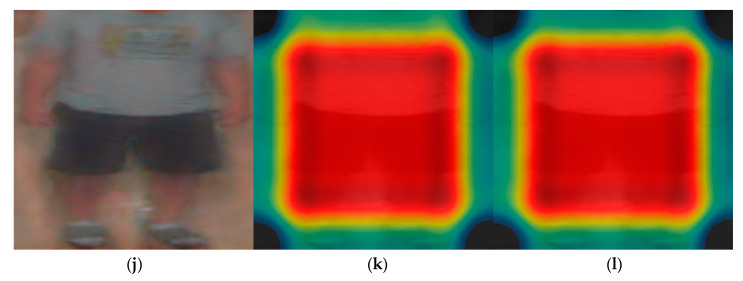
Results on class activation feature map on DFB-DB2. (**a**,**d**) Input face images, (**b**,**c**,**e**,**f**) results via VGG face net-16 in rectified linear units (ReLU) layer, (**b**,**e**) images from 7th ReLU layer, (**c**,**f**) images from 13th ReLU layer, (**g**,**j**) input body images, (**h**,**i**,**k**,**l**) results via ResNet-50 in batch normalized layer, (**h**,**i**) images from last batch normalized layer on conv5 2nd block, (**k**,**l**) images from last batch normalized layer on conv5 3rd block.

Subsequently, we analyzed the class activation feature map of VGG face net-16 and ResNet-50 that were used for the DFB-DB2 to evaluate the recognition performance for face and body images. Figure 13 shows the class activation feature map from a specific layer using Grad-CAM method [48]. Furthermore, the important features shown through the distribution. Figure 13a,d,g,j correspond to the input face and body images of the CNN model, and Figure 13b,c,e,f,h,i,k,l show the class activation feature map results of face and body images.

Specifically, when the input (a) is processed through VGG face net-16, (b) corresponds to the class activation feature map of the 7th ReLU layer, and (c) corresponds to the class activation feature map of the 13th ReLU layer. The image in (c) shows the distribution focused around the face area where the red color represents the main feature, while the blue color represents less important features. The black color indicates that no features were detected. When the process goes from (b) to (c), the features are more focused around the face region. Additionally, body images were extracted from the batch normalized layer. In contrast to the face image results, the main features were observed around the body region because the trained part of the ResNet-50 model considers information with respect to the individual’s body and clothes as important features.

#### 5.3.3. Testing with CNN Model for ChokePoint Dataset

##### Ablation Study

**Figure 14:**
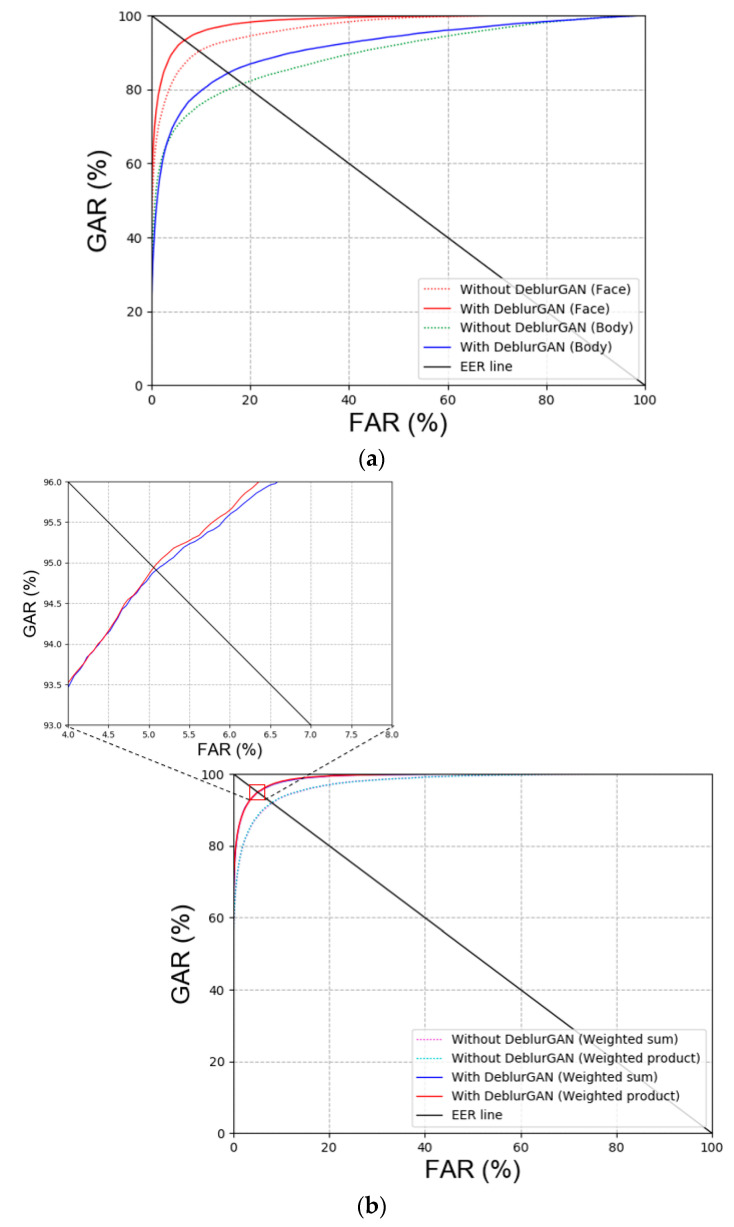
ROC curves with and without DeblurGAN performance on ChokePoint dataset. (**a**) Face and body image recognition results and (**b**) score-level fusion result.

The images restored with DeblurGAN and images with a score exceeding the threshold value were combined in the experiment, as proposed in the study. Based on the results in Table 10 and Table 11, the weighted sum method, among the score-level fusion methods, exhibited better results. Figure 14 shows the results in Table 10 and Table 11 in the form of plots. As shown in the plots in Figure 14, the recognition performance improves when DeblurGAN is applied. Furthermore, the weighted product method exhibited better results among score-level fusion methods.

##### Comparison between Previous Methods and Proposed Method

**Figure 15:**
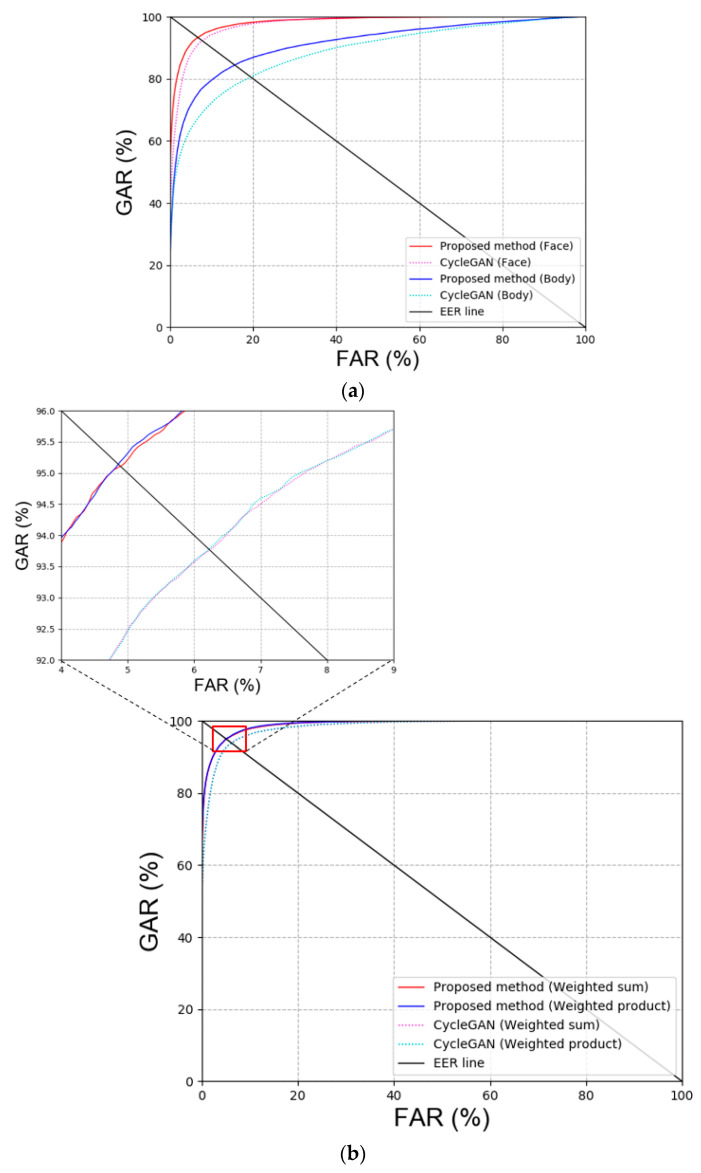
ROC curves for the proposed and CycleGAN in ChokePoint dataset. (**a**) Face and body recognition result and (**b**) score-level fusion result.

With respect to the GAN models for blur image restoration, the performance of CycleGAN and DeblurGAN was compared. Table 12 and Figure 15 show the results and plots, respectively. The results indicated that DeblurGAN exhibited better recognition performance than CycleGAN.

**Figure 16:**
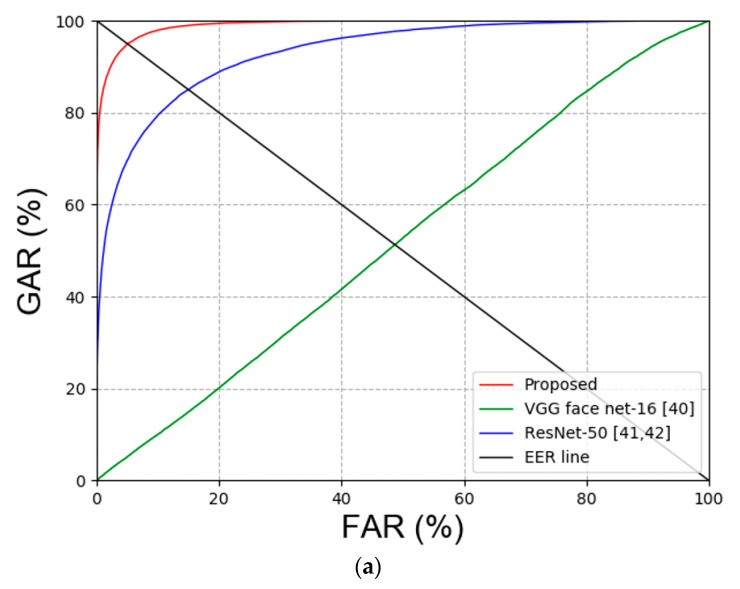

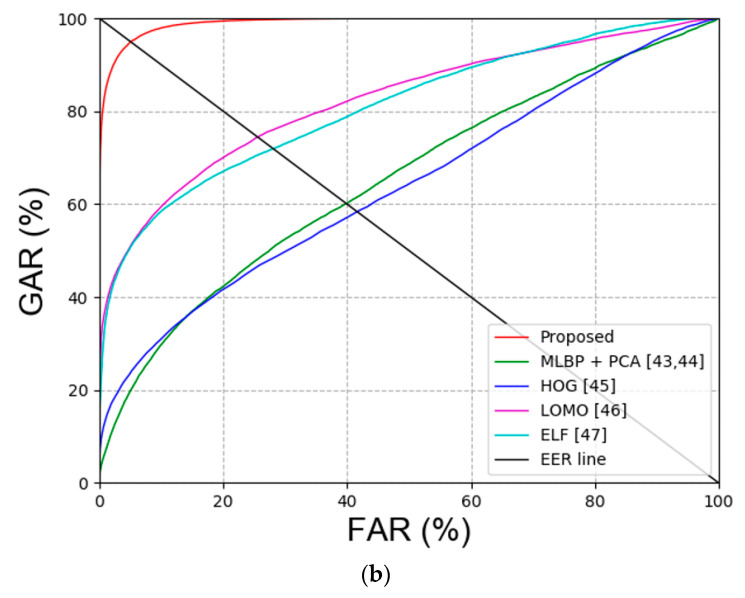
ROC curves for proposed and the state-of-art-method on ChokePoint dataset. (**a**) Face image results and (**b**) face and body image results.

Second, the existing face recognition and face and face and body recognition methods were compared with the proposed method. Table 13 and Table 14 show the experimental results, and Figure 16 illustrates the results in the plots.

**Figure 17:**
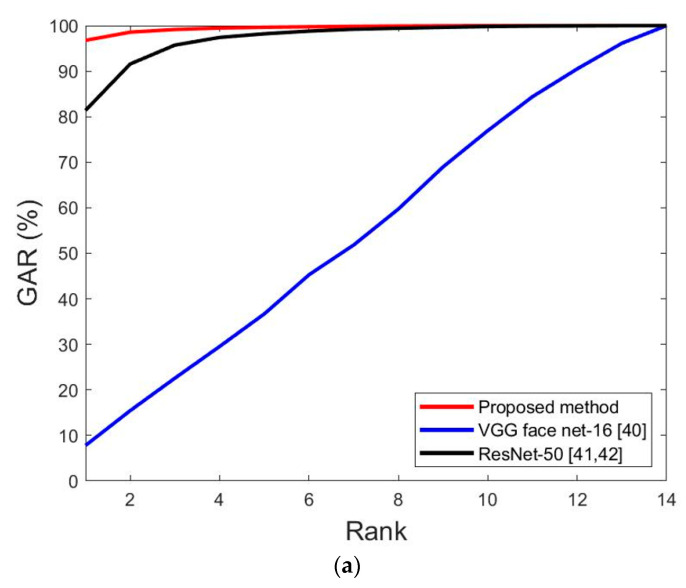

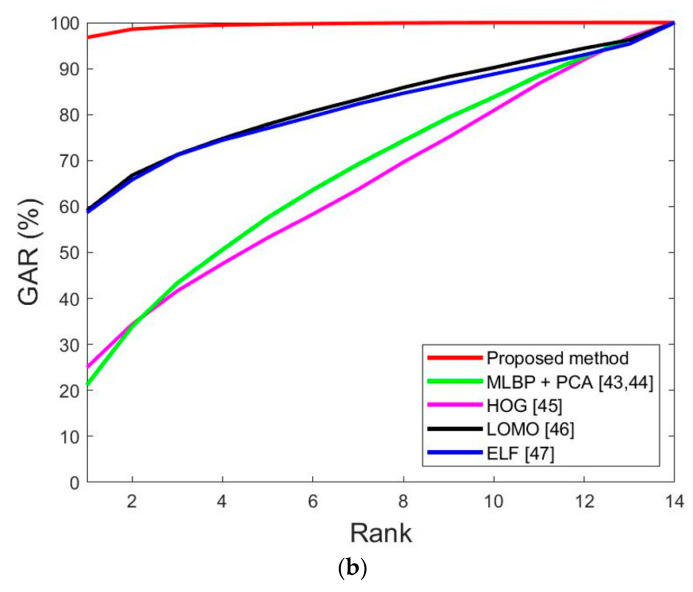
CMC curves for the proposed and previous method on ChokePoint dataset. (**a**) Face image recognition results via the proposed and previous methods; (**b**) face and body image recognition results for the proposed and previous methods.

Figure 17 shows the comparison of the CMC curve of the proposed method and previous methods for face and face and body recognition. As shown in Figure 17a,b, the performance of the proposed method exceeded that of other methods.

**Figure 18:**
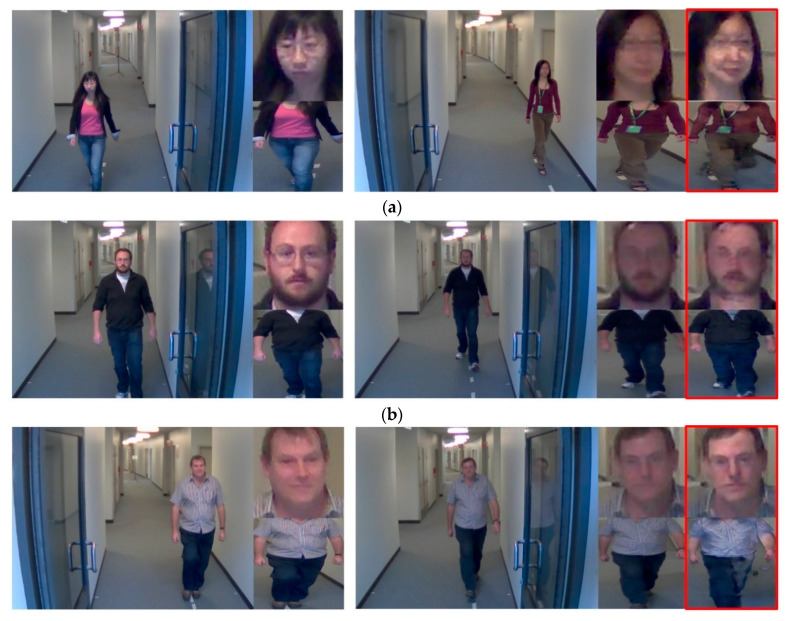

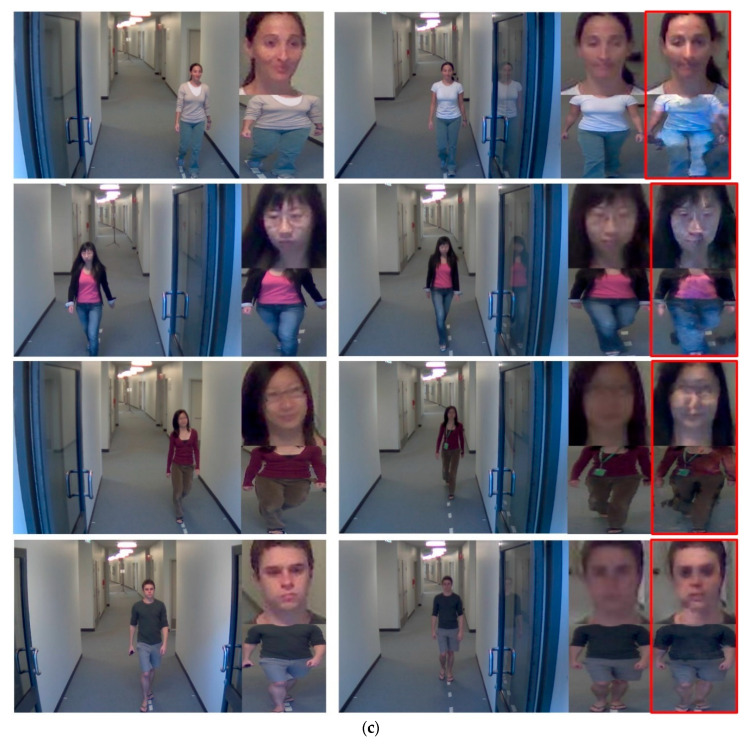
Cases corresponding to false acceptance (FA), false rejection (FR), and correction recognition (**a**)–(**c**) Cases from ChokePoint dataset (**a**) FA cases, (**b**) FR cases, and (**c**) cases of correct recognition.

The results of the proposed method using the ChokePoint dataset are shown for the cases of FAR, FRR, and correct recognition in Figure 18.

**Figure 19:**
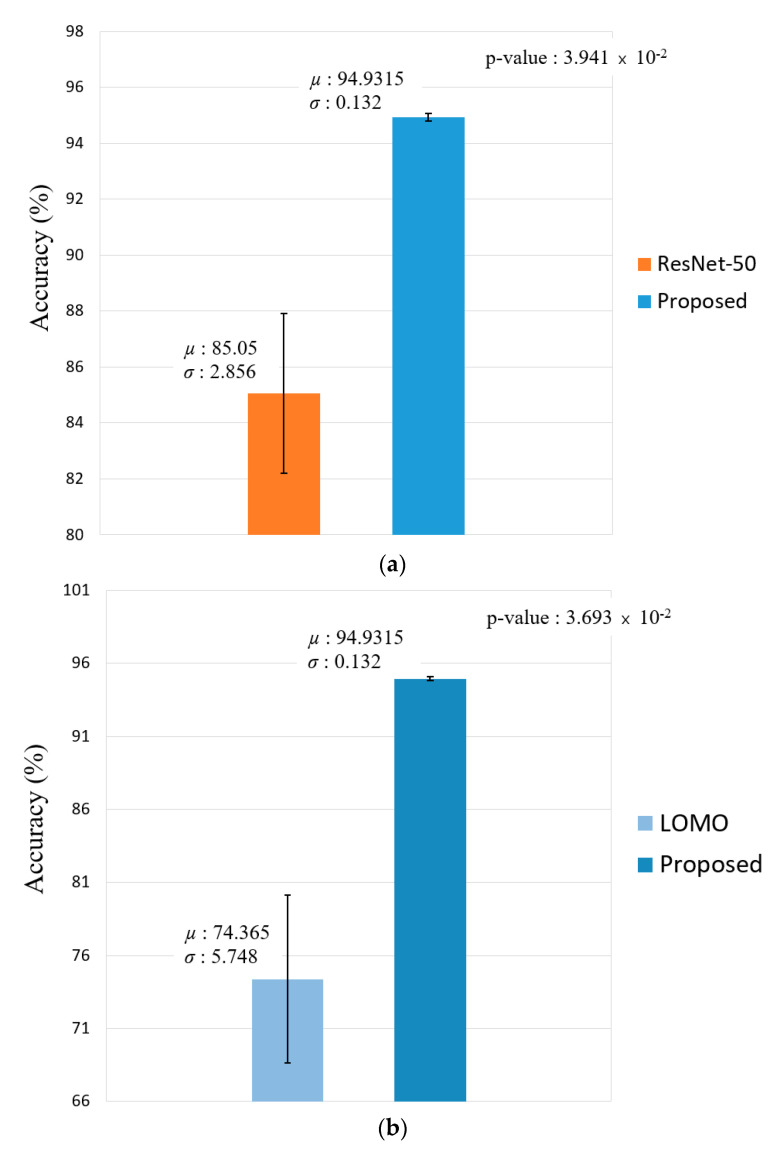
T-test performance of the proposed method and second-best model in terms of average accuracy. (**a**) Comparison of the proposed method and ResNet-50 and (**b**) comparison of the proposed method and local maximal occurrence (LOMO).

Figure 19 shows the difference in the performance by measuring the Cohen’s d-value and t-test results of face recognition and face and body recognition and comparison with the proposed method. With respect to face recognition, the Cohen’s d-value between the proposed method and ResNet-50 [41,42] is 4.89, and this significantly exceeds the effect size of 0.8, thus its being high. The *p*-value of the t-test is approximately 0.03941, which differs from the proposed method by 99.961%. With respect to face and body recognition, Cohen’s d-value and t-test results were measured for the ELF [47] that exhibited the second-best performance when compared to that of the proposed method. The Cohen’s d-value is 5.06, thereby exhibiting a large effect size, and the t-test exhibited a difference of 99.963%.

#### 5.3.4. Class Activation Map

**Figure 20:**
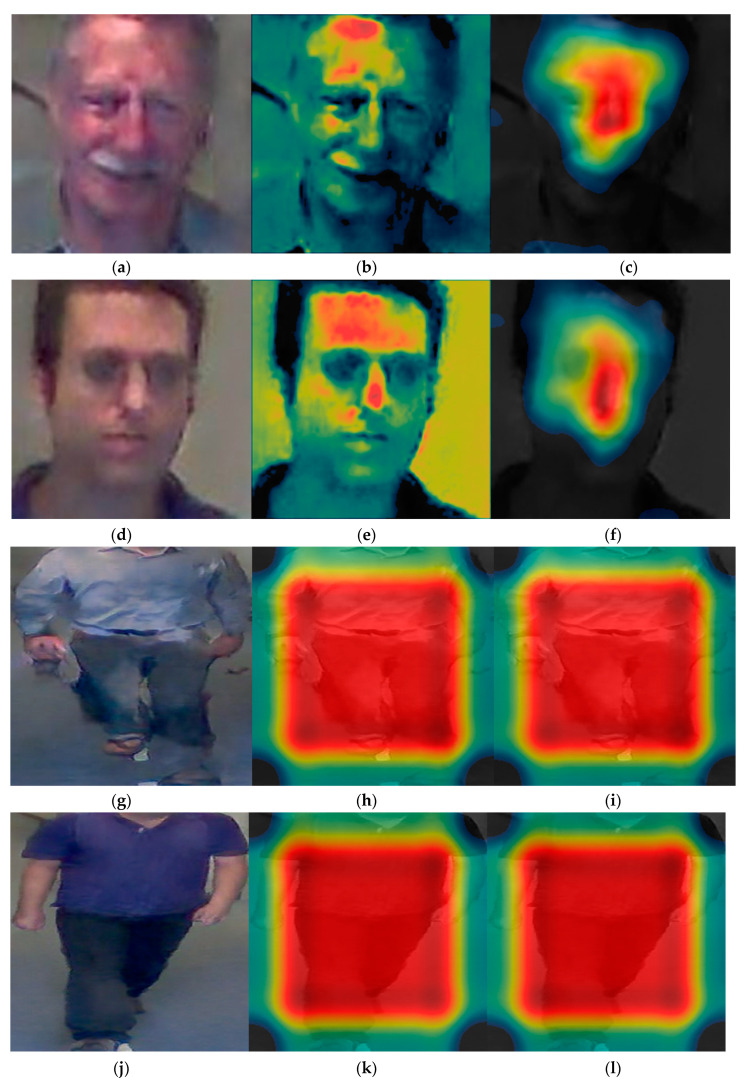
Result on class activation feature map on ChokePoint dataset. (**a**,**d**) Input face images, (**b**,**c**,**e**,**f**) results for the VGG face net-16 in ReLU layer, (**b**,**e**) images from 7th ReLU layer, (**c**,**f**) images from 13th ReLU layer, (**g**,**j**) input body images, (**h**,**i**,**k**,**l**) results for the ResNet-50 in batch normalized layer, (**h**,**i**) images from the last batch normalized layer on conv5 2nd block, and (**k**,**l**) images from the last batch normalized layer on conv5 3rd block.

In the subsequent experiment, the class activation feature map of the ChokePoint dataset was examined. Figure 20 shows the class activation feature map results. The face image signifies the class activation feature map obtained from the ReLU layer of VGG face net-16. Figure 20h,i,k,l of Figure 20b,c,e,f body image represent the class activation feature map of the image that passed through the batch normalized layer. In the result of the images, the red color represents the main feature, and the blue color represents less important features. Similar results to the experiment using DFB-DB2 are obtained in Figure 20.

#### 5.3.5. Comparisons of Processing Time on Jetson TX2 and Desktop Computer

**Figure 21:**
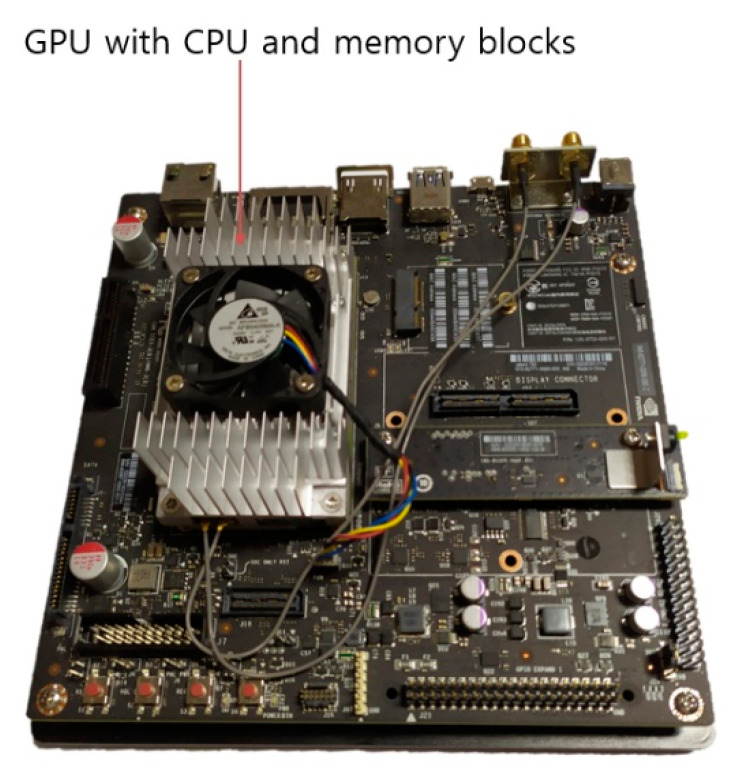
Jetson TX2 embedded system.

In the next experiment, the computing speed of the proposed method was compared using Jetson TX2 board [49] as shown in Figure 21 and a desktop computer including NVIDIA GeForce GTX 1070 graphic processing unit (GPU) card. Jetson TX2 board is an embedded system equipped with NVIDIA Pascal™ GPU architecture with 256 NVIDIA CUDA cores, 8 GB 128-bit LPDDR4 memory, and dual-core NVIDIA Denver 2 64-Bit CPU. The power consumption is less than 7.5 watts. The proposed method was ported with Keras [50] and TensorFlow [51] in Ubuntu 16.04 OS. The versions of the installed framework and library include Python 3.5 and TensorFlow 1.12; NVIDIA CUDA^®^ toolkit [52] and NVIDIA CUDA^®^ deep neural network library (CUDNN) [53] versions are 9.0 and 7.3, respectively.

As shown in Table 15 and Table 16, our method requires the time cost of a total of 75.72 ms and 481.7 ms on desktop computer and Jetson TX2 embedded system, respectively, which means that our method can be operates at the speed of 13.2 frames/s (1000/75.72) and 2.08 frames/s (1000/481.7) on desktop computer and Jetson TX2 embedded system, respectively. The Jetson TX2 embedded system has less computing resource and GPU of lower speed compared to those in the desktop computer. Therefore, the processing speed on Jetson TX2 is slower than that on desktop computer. However, more advanced and cheaper GPU card and embedded GPU system have been fast commercialized, and our method can be operated at faster speed on those systems.

## 6. Conclusions

There were lots of works that use GAN for deblur [38,39,54,55,56]. However, most previous works aimed at the visibility enhancement of general scene images, whereas the main purpose of our research is to enhance the recognition accuracy of face and body images. In the previous works, GAN tried to generate the image of high visibility and distinctiveness although limited amount of noise is additionally included in the generated image. However, GAN in our research tries to generate the face and body images with which the higher recognition accuracies can be obtained. It means that the maximization of intra-class consistency (from matching between same people) and inter-class variation (from matching between different people) in the generated image is more important than the visibility enhancement in our GAN. Therefore, we compared the recognition accuracies of face and body images by our GAN with those by other GANs, as shown in Table 7 and Table 12 and Figure 8 and Figure 15, instead of the metrics showing the image visibility, such as peak signal-to-noise ratio (PSNR) and structural similarity (SSIM), like previous works [38,39,54,55,56]. Consequently, it is not appropriate to use our method to handle the natural images.

The study proposed a deep CNN-based recognition method involving a score-level fusion approach for face and body images in which a GAN is applied to restore the blur problem that is generated when body recognition data is obtained in indoor environments from a long distance. Previous studies focused on minimizing a blur if discovered in face images although deblurring is typically omitted for body images because detailed information is considered as absent in body images when compared to the face images. However, the blur problem in body images affects recognition performance. To solve the problem, face images and body images were separated, and a blur was then restored using a GAN model in the study. Higher processing time is obtained if restoration is performed independently for face and body images using a GAN model. However, better restoration of distinctive features of face and body is observed. For impartial comparison experiments, the GAN model was used for restoration, VGG face net-16 and ResNet-50 were used for training in the study, and the DFB-DB2 built by the researchers was disclosed.

In future work, we would research about the advanced GAN model which can process the face and body images simultaneously. For that, we also consider the scheme of pre-classification of input image into face and body image, as well as adopting different loss functions according to input image. In addition, we would study the combined structure of GAN and recognition CNN models for the reduction of training time, and the measures to increase the processing speed of an embedded system would be explored via examining a lighter GAN for deblurring. Furthermore, our deblur-based recognition method would be applied to various biometric systems, including iris and finger-vein to evaluate recognition performance.

## Figures and Tables

**Table 1:** Summary of this study and previous studies on person recognition using surveillance camera environment.

Category			Method	Advantage	Disadvantage
Without blur restoration	Single modality	Face recognition	PCA [1]	Low performance degradation due to lighting changes.	Degraded recognition performance due to distortion of image features due to a blur.
			SML-MKFC with DA [2]		
		Texture and shape-based body recognition	S-CNN [4]	Low interference of a blur for recognition.	Different individuals wearing the same clothes are recognized as the same individual.
			CNN + PCA, SVM [5]		
			CNN + DDML [6]		
		Movement-based body recognition	PCA + MDA [3]	Less affected directly by a blur.	Requires an extensive time for acquiring data.
	Multimodal	Movement-based body and face recognition	HMM/Gabor features based EBGM [7]	Less affected by a blur because it is gait-based body recognition.	Sufficient data is required for continuous images. Distortion of face image due to noise, such as lighting changes or blur.
			Eigenface calculation and αGEI [8]		
		Texture and shape-based body and face recognition	VGG face net-16 and ResNet-50 [9]	Easy to acquire data because continuous images are not required.	Sufficient data is required for finetuning based on data characteristics.
With blur restoration	Single modality	Face recognition	Fast TV-l1 and Deconvolution+ PCA [11]	Improved recognition performance, as blurred face images are restored.	Most studies focused on comparing the deblurred facial image with the original image.
			DeblurLPQ [12]		
			Wien filters or BTV regularization [13]		
			CSR + ASDS-AR [14]		
			PSF + Wiener filter [15]		
			UMSN [16]		
	Multimodal	Texture and shape-based body and face recognition	Proposed method	Improved performance because body and face were separated for restoration.	Slow processing time due to restoration is performed twice for body and face.

**Table 2:** Generator of DeblurGAN. GAN = generative adversarial network.

Layer Type		Size of Feature Map	Number of Filters	Size of Filters	Number of Strides	Number of Iterations
Image input layer		256 (height) × 256 (width) × 3 (channel)				
Convolution layer		256 × 256 × 64	64	7 × 7	1	
Instance normalization layer						
ReLU						
Convolution block 1	Convolution layer	128 × 128 × 128	128	3 × 3	2	
	Instance normalization layer					
	ReLU					
Convolution block 2	Convolution layer	64 × 64 × 256	256	3 × 3	2	
	Instance normalization layer					
	ReLU					
Resblocks	Convolution layer	64 × 64 × 256	256	3 × 3	1	9
	Instance normalization layer					
	ReLU					
	Convolution layer	64 × 64 × 256	256	3 × 3	1	
	Instance normalization layer					
Transposed blocks 1	Contraposed layer	128 × 128 × 128	128	4 × 4	2	2
	Instance normalization layer					
	ReLU					
Transposed blocks 2	Contraposed layer	256 × 256 × 64	64	4 × 4	2	
	Instance normalization layer					
	ReLU					
Convolution layer (Output layer)		256 × 256 × 3	3	7 × 7	1	

**Table 3:** Discriminator of DeblurGAN (All convolution layers 1–5 * indicate that they have two paddings.).

Layer Type	Size of Feature Map	Number of Filters	Size of Filters	Number of Strides
Input image	256 × 256 × 3			
Target image	256 × 256 × 3			
Concatenator	256 × 256 × 6			
Convolution layer1 *	129 × 129 × 64	64	4 × 4 × 6	2
Convolution layer2 *	65 × 65 × 128	128	4 × 4 × 64	2
Convolution layer3 *	33 × 33 × 256	256	4 × 4 × 128	2
Convolution layer4 *	34 × 34 × 512	512	4 × 4 × 256	1
Convolution layer5 * (Output layer)	35 × 35 × 1	1	4 × 4 × 512	1

**Table 4:** Total images of DFB-DB2 and ChokePoint dataset.

		DFB-DB2			Chokepoint Dataset		
		Number of Classes in Each Fold	Number of Augmented Images (For Training)	Number of Images (For Testing)	Number of Classes in Each Fold	Number of Augmented Images (For Training)	Number of Images (For Testing)
Face	Sub-Dataset1	11	200,134	827	14	519,050	10,381
	Sub-Dataset2	11	239,338	989	14	513,450	10,269
Body	Sub-Dataset1	11	200,134	827	14	519,050	10,381
	Sub-Dataset2	11	239,338	989	14	513,450	10,269

**Table 5:** Comparison of equal error rate (EER) for face recognition and body recognition on DFB-DB2 without or with DeblurGAN (unit: %).

Method		Face	Body
Without DeblurGAN (without focus score checking)	1st fold	45.29	33.68
	2nd fold	41.85	28.7
	Average	43.52	31.19
With DeblurGAN (with focus score checking)	1st fold	12.44	21
	2nd fold	7.94	16.48
	Average	10.19	18.74

**Table 6:** Comparison of EER for score-level fusion on DFB-DB2 without or with DeblurGAN (unit: %).

Method		Score-Level Fusion	
		Weighted Sum	Weighted Product
Without DeblurGAN (without focus score checking)	1st fold	32.44	32.57
	2nd fold	27.94	27.93
	Average	30.19	30.25
With DeblurGAN (with focus score checking)	1st fold	9.835	9.901
	2nd fold	5.552	5.557
	Average	7.694	7.729

**Table 7:** Comparisons of EER for recognition by proposed method with those by other GAN-based methods in DFB-DB2 (unit: %).

Method		Average
Face	Proposed method	10.19
	CycleGAN [20]	13.255
	Pix2pix [22]	13.315
	AGGAN [37,38]	23.01
	DeblurGANv2 [39]	15.64
Body	Proposed method	18.74
	CycleGAN [20]	15.745
	Pix2pix [22]	23.195
	AGGAN [37,38]	22.52
	DeblurGANv2 [39]	22.71
Weighted sum	Proposed method	7.694
	CycleGAN [20]	8.29
	Pix2pix [22]	11.49
	AGGAN [37,38]	14.649
	DeblurGANv2 [39]	11.801
Weighted product	Proposed method	7.729
	CycleGAN [20]	8.41
	Pix2pix [22]	11.5605
	AGGAN [37,38]	14.342
	DeblurGANv2 [39]	11.869

**Table 8:** Comparison of EER for the results of the proposed method and previous face recognition methods (unit: %).

Method	1st Fold	2nd Fold	Average
Proposed method	9.835	5.552	7.694
VGG face net-16 [40]	17.44	16.71	17.075
ResNet-50 [41,42]	13.96	14.06	14.01

**Table 9:** Comparison of EER for the results of the proposed and previous face and body recognition methods (unit: %).

Method	1st Fold	2nd Fold	Average
Proposed method	9.835	5.552	7.694
MLBP + PCA [43,44]	29.38	27.84	28.61
HOG [45]	38.09	44.14	41.12
LOMO [46]	21.98	23.4	22.69
ELF [47]	20.91	23.87	22.39

**Table 10:** Comparison of EER for face recognition and body recognition on ChokePoint dataset without or with DeblurGAN (unit: %).

Method		Face	Body
Without DeblurGAN (without focus score checking)	1st fold	11.76	18.50
	2nd fold	8.09	18.46
	Average	9.925	18.48
With DeblurGAN (with focus score checking)	1st fold	7.05	15.97
	2nd fold	6.39	15.2
	Average	6.72	15.585

**Table 11:** Comparison of EER for score-level fusion on ChokePoint dataset without or with DeblurGAN (unit: %).

Method		Score-Level Fusion	
		Weighted Sum	Weighted Product
Without DeblurGAN (without focus score checking)	1st fold	9.84	9.79
	2nd fold	6.55	6.43
	Average	8.195	8.11
With DeblurGAN (with focus score checking)	1st fold	5.162	5.163
	2nd fold	4.99	4.975
	Average	5.076	5.069

**Table 12:** Comparisons of EER for recognition by proposed method with that by CycleGAN (unit: %).

Method		1st Fold	2nd Fold	Average
Face	Proposed method	7.05	6.39	6.72
	CycleGAN [20]	9.05	6.43	7.74
Body	Proposed method	15.97	15.2	15.585
	CycleGAN [20]	18.41	20.41	19.41
Weighted sum	Proposed method	5.162	4.99	5.076
	CycleGAN [20]	7.05	5.331	6.1905
Weighted product	Proposed method	5.163	4.975	5.069
	CycleGAN [20]	7.023	5.362	6.1925

**Table 13:** Comparison of EER for recognition results via the proposed method and previous face recognition methods (unit: %).

Method	1st Fold	2nd Fold	Average
Proposed method	5.163	4.975	5.069
VGG face net-16 [40]	49.29	48.25	48.77
ResNet-50 [41,42]	12.93	16.97	14.95

**Table 14:** Comparison of EER for recognition results via the proposed and previous face and body recognition methods (unit: %).

Method	1st Fold	2nd Fold	Average
Proposed method	5.163	4.975	5.069
MLBP + PCA [43,44]	37.75	42.38	40.07
HOG [45]	41.84	41.47	41.66
LOMO [46]	29.7	21.57	25.635
ELF [47]	31.93	23.94	27.935

**Table 15:** Comparison of processing time on Jetson TX2 and desktop computer by DeblurGAN (unit: ms).

Platform	DeblurGAN
Desktop computer	32
Jetson TX2	349

**Table 16:** Comparison of processing time on Jetson TX2 and desktop computer by VGG face net-16 and ResNet-50 (unit: ms).

Platform	VGG Face Net-16	ResNet-50	Total
Desktop computer	24.8	18.92	43.72
Jetson TX2	91.9	40.8	132.7
